# Insulin-like factor regulates neural induction through an IGF1 receptor-independent mechanism

**DOI:** 10.1038/srep11603

**Published:** 2015-06-26

**Authors:** Yoshikazu Haramoto, Shuji Takahashi, Tomomi Oshima, Yasuko Onuma, Yuzuru Ito, Makoto Asashima

**Affiliations:** 1 Research Center for Stem Cell Engineering, National Institute of Advanced Industrial Science and Technology (AIST), Tsukuba Central 4, 1-1-1 Higashi, Tsukuba, Ibaraki 305-8562, Japan; 2 Institute for Amphibian Biology, Graduate School of Science, Hiroshima University, 1-3-1 Kagamiyama, Higashi-Hiroshima, Hiroshima 739-8526, Japan; 3 Center for Structuring Life Sciences, Graduate School of Arts and Sciences, The University of Tokyo, 3-8-1 Komaba, Meguro-ku, Tokyo 153-8902, Japan

## Abstract

Insulin receptor (IR) and insulin-like growth factor-1 receptor (IGF1R) signalling is required for normal embryonic growth and development. Previous reports indicated that the IGF/IGF1R/MAPK pathway contributes to neural induction and the IGF/IGF1R/PI3K/Akt pathway to eye development. Here, we report the isolation of *insulin3* encoding a novel insulin-like ligand involved in neural induction. Insulin3 has a similar structure to pro-insulin and mature IGF ligands, but cannot activate the IGF1 receptor. However, similar to IGFs, Insulin3 induced the gene expression of an anterior neural marker, *otx2*, and enlarged anterior head structures by inhibiting Wnt signalling. Insulin3 are predominantly localised to the endoplasmic reticulum when *otx2* is induced by insulin3. Insulin3 reduced extracellular Wnts and cell surface localised Lrp6. These results suggest that Insulin3 is a novel cell-autonomous inhibitor of Wnt signalling. This study provides the first evidence that an insulin-like factor regulates neural induction through an IGF1R-independent mechanism.

Insulin family members have significant roles as bioactive substances. Insulin controls blood glucose levels, and insulin-like growth factor (IGF) influences growth, differentiation, and survival of cells. IGF signals are transduced through the type I IGF receptor (IGF1R), which is closely related to the insulin receptor (IR). IGF1R and IR are receptor tyrosine kinases (RTKs) and activate the PI3K/Akt and mitogen-activated protein kinase (MAPK) signalling pathways^1^. Previous studies suggest that the two pathways are required for different events early in development. The IGF/IGF1R/PI3K/Akt pathway is essential for eye development and several molecules involved in this step have been identified. Kermit2, an IGF receptor binding protein, and B56ε regulatory subunit of protein phosphatase 2A (PP2A) are required for the IGF/IGF1R/PI3K/Akt pathway and eye development^2^,^3^. It has been reported that the IGF/IGF1R/MAPK pathway is important for neural induction. In *Xenopus*, neural tissue can be induced by RTKs via MAPK^4^. MAPK phosphorylates the linker region of Smad1, a BMP signal mediator, and attenuates BMP signals by degradation of Smad1 protein, resulting in the induction of neural tissues^5^,^6^. The expressions of *otx2* and *sox3*, early neural marker genes, induced by IGF were not affected by co-injection of *Δp85*, a dominant negative regulatory subunit of PI3K, *dnAkt*, dominant negative Akt, or treatment of LY294002, a chemical PI3K inhibitor. However, expression of an eye field transcription factor, *rx*, was reduced^2^. Thus, eye development and neural induction require different IGF signalling.

Previous studies revealed IGFs can inhibit Wnt signalling^6^,^7^. *IGF* over-expressing embryos resemble the typical phenotype of Wnt inhibition, which develops a giant cement gland. Head formation and neural induction by IGF is required for Wnt inhibition and is mediated by *otx2* induction^7^,^8^. *Otx2* is a homeobox gene that demarcates and specifies anterior neural regions^8^. Wnt signals inhibit GSK3β and induce the accumulation of β-catenin, resulting in activation of down-stream target gene expression. The IGF/IGF1R/PI3K/Akt pathway also inhibits GSK3β and activates Wnt signalling^9^. These findings suggest *otx2* induction and Wnt inhibition by IGF1 is mediated through IGF1R/MAPK pathway, not IGF1R/PI3K/Akt pathway^2^,^5^,^6^.

Various molecules secreted from dorsal mesoendoderm act as dorsalising and neural inducing factors by inhibiting caudalising factors, BMPs and Wnts, and thereby protect the anterior neural region where *otx2* is expressed. These molecules include BMP inhibitors, Noggin, Chordin, Follistatin, a Wnt inhibitor Dickkopf-1 (Dkk1), and a multipotent inhibitor, Cerberus^10^,^11^,^12^,^13^,^14^. These Wnt inhibitors secreted from the mesodermal organizer interact with their targeting ligands or receptors in the extracellular space in a non-cell-autonomous manner, and act as a watchdog to protect the head region.

A previous study showed that focal adhesion kinase (FAK) regulates *wnt3a* expression to balance anterior-posterior cell fate specification in the developing neural plate of *Xenopus*^15^. *FAK* knockdown induces giant cement gland and anteriorises the embryo in early *Xenopus* development. This anteriorised phenotype resembles that of embryos with zygotic knock down of canonical Wnt signalling. Indeed, *wnt3a* expression is strongly inhibited at all stages in *FAK* morphants, while *wnt8* expression is normal^15^. Thus, the ligand-specific inhibition of Wnts is sufficient to anteriorise *Xenopus* embryos.

Few factors have been reported to be intracellular regulators of extracellular components, ligands or receptors, of Wnt signals. Porcupine regulates N-glycosylation and transportation of Wnt ligand^16^,^17^,^18^. Mesd functions as a chaperone protein for Lrp5/6 that is required for transport of coreceptors to cell surfaces^19^,^20^. Shisa interacts with immature forms of the Wnt receptor Frizzled within the endoplasmic reticulum (ER), and suppresses its maturation and trafficking to the cell surface. It is unclear whether Wnt8 and Wnt3a have regulators that function in a cell-autonomous manner in the posterior neuroectoderm of *Xenopus* embryos.

Here, we report the isolation of *insulin3* encoding a novel insulin-like ligand involved in anterior neural development. This factor has a similar structure to mature IGF ligands, and is expressed at the presumptive neuroectoderm at early gastrulae and posterior neuroectoderm at neurulae. *Insulin3* loss-of-function experiments in both *X. laevis* and *X. tropicalis* embryos revealed it is an essential factor for head formation. Insulin3 can inhibit canonical Wnt signalling and over-expressed Insulin3 is not efficiently secreted into the extracellular space when *otx2* is already induced. Furthermore, *otx2* induction by Insulin3 is not mediated through the IGF1R/MAPK pathway. Insulin3 suppresses Wnt co-receptor Lrp maturation and localisation to the cell surface and reduces the total amount of Wnt ligands. These results indicate possibility that Insulin3 is a novel cell-autonomous inhibitor of Wnt signalling. This study provides the first evidence that an insulin-like factor regulate neural induction through an IGF1R-independent mechanism.

## Results

We cloned a novel insulin-like factor. Almost all vertebrates have two insulin-like growth factors, *IGF1* and *IGF2*. *IGF2* and *insulin* are located as a tandem repeat in the genome. It was previously demonstrated that *Xenopus* has a third insulin-like growth factor loci, *IGF3*^6^. In the current study, we identified another insulin-like factor located next to the *IGF3* loci (Supplementary Fig. S1). By phylogenetic analysis, the open reading frame (ORF) of *insulin* consists of 1–2 exons. *IGF1* and *IGF2* ORFs consist of 4–6 and 3–4 exons, respectively. *Xenopus*-specific *IGF3* ORF consists of 4 exons similar to other *IGFs*. The novel insulin-like factor ORF reported here consists of 2 exons similar to *insulin*. Phylogenetic analysis using protein sequences indicated that this molecule could be classified in the Insulin group, not the IGF group or Relaxin group (Supplementary Fig. S2a). Thus, phylogenetic analyses indicated this new factor should be called Insulin3. Insulin3 consists of 100 amino acids (UGID: 748710). Amino acid sequence alignment between Insulin and Insulin3 indicates that Insulin3 has 6 cysteine residues conserved among the Insulin family, and no proteolytic cleavage sites that are required for the release of C-peptide (Supplementary Fig. S2b). Western blot analysis also showed that Insulin3 is not proteolytically processed to release C-peptide (Supplementary Fig. S2c). These results suggested the protein structure of Insulin3 corresponded to pro-Insulin or mature IGFs (Supplementary Fig. S2c).

**Figure 1:**
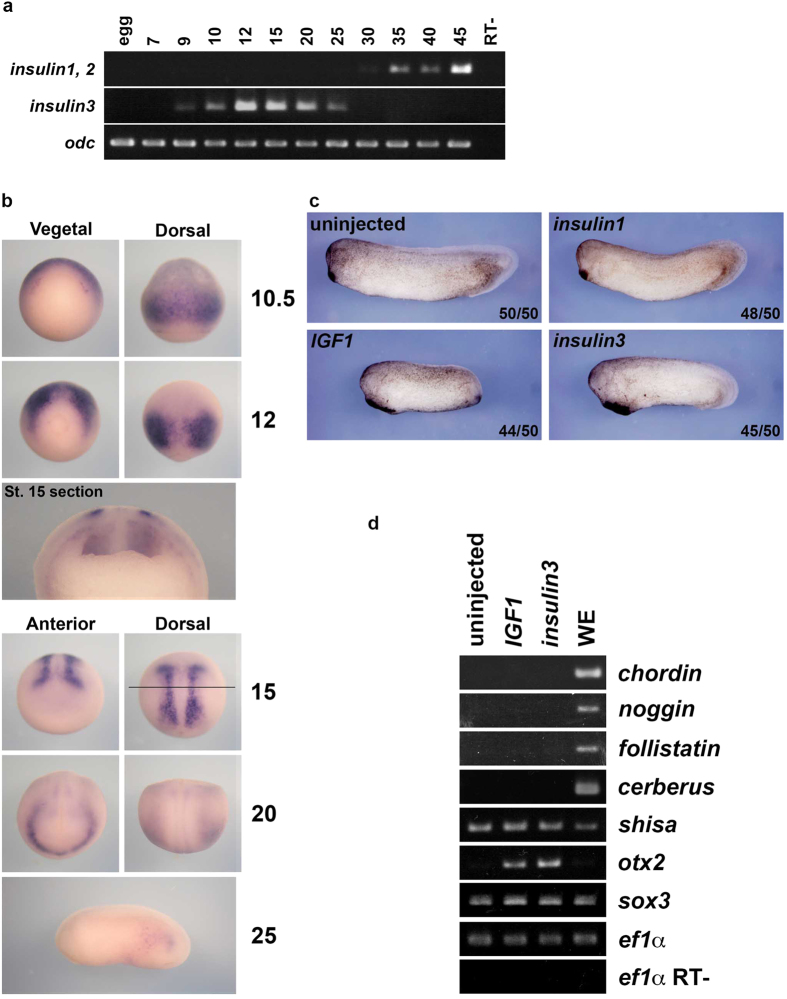
Characterization of *insulin3* in *Xenopus*. (**a**) Stage PCR for *insulin3*. Stages of samples are indicated (top). (**b**) Whole-mount *in situ* hybridization of *insulin3* transcripts. Stages of samples are indicated (right). Viewpoints are indicated (top). The line indicates the position of the cross-section. (**c**) mRNAs were injected into the animal pole of two-cell-stage *X*. *laevis* embryos. Embryos were harvested at stage 30. Amounts of mRNA injected per embryos were: *insulin1* (2 ng), *IGF1* (300 pg) and *insulin3* (300 pg). Expanded cement gland was induced by injection of *insulin3* and *IGF1*, but not by injection of *insulin1*. (**d**) Animal cap assay. One ng of *IGF1* and *insulin3* mRNAs were injected into the animal pole of both blastomeres at the two-cell stage in *X*. *laevis* embryos. Animal caps were dissected at stage 9 and harvested at stage 11. IGF1 and Insulin3 induce *otx2* and inhibit without induction of BMP antagonists. WE: whole embryo. RT-: reverse transcriptase minus reaction. Full-length gels are presented in Supplementary figure S7.

*Insulin3* is expressed from late blastulae to the early tailbud stage (Fig. 1a). At the early gastrulae, *Insulin3* was detected in whole presumptive neuroectoderm (Fig. 1b). At the late gastrulae and the early neurulae stage (stage 15), *insulin3* expression was detected at the midpoint of the mediolateral axis of the neural plate and slightly at the archenteron roof. The *insulin3* expressing cells are located in the subepithelial layer of the neural plate. At the late neurulae stage (stage 20), *insulin3* expression appears in the anterior endoderm forward of the prechordal plate (Fig. 1b).

Over-expression of *insulin3* mRNA in the animal pole at the 2-cell stage enlarged the anterior structure with giant cement gland as well as *IGF1*, whereas over-expression of *insulin* does not (Fig. 1c). Insulin3 and IGF1 induce the expression of an anterior neural marker, *otx2*, in stage 11 animal caps without changing the expressions of BMP antagonists, *chordin*, *noggin*, *follistatin*, and *cerberus*, and a Wnt and FGF inhibitor, *shisa* (Fig. 1d).

**Figure 2:**
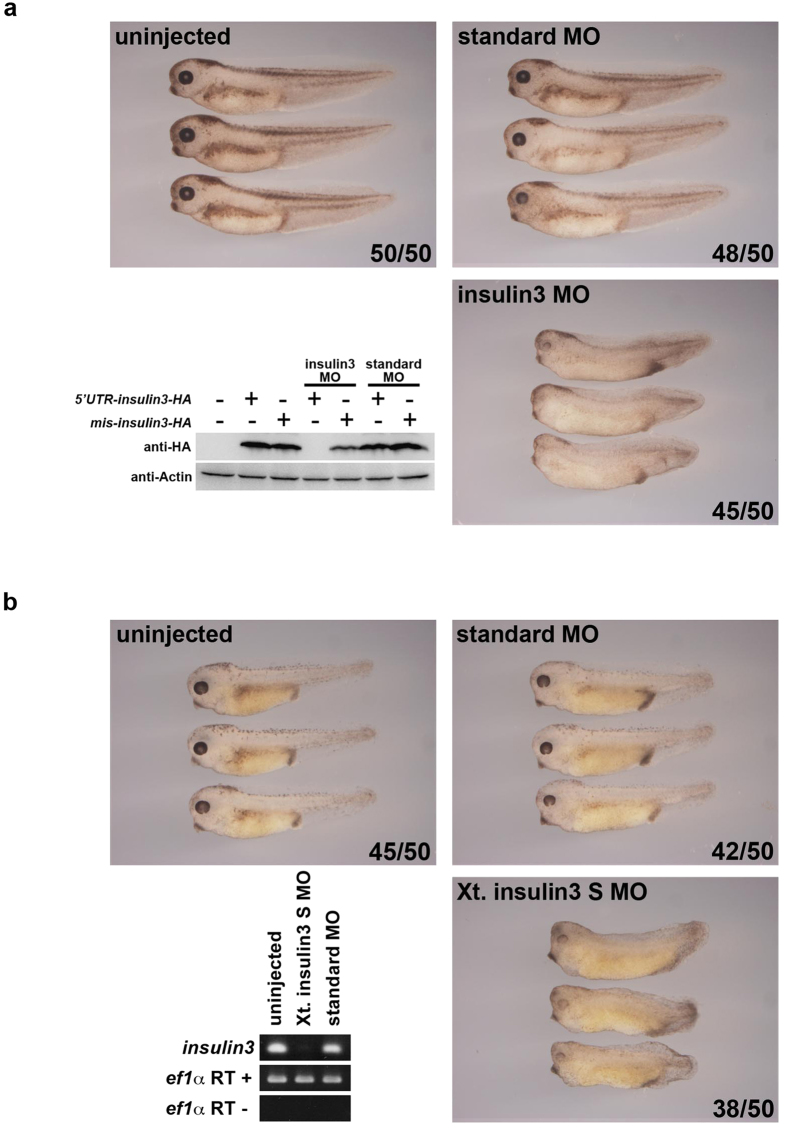
*Insulin3*-deficient embryos showed anterior defects in both X. *laevis* and X. *tropicalis*. A translation-inhibiting MO was designed for *X. laevis insulin3* and a splice-inhibiting MO for *X. tropicalis insulin3*. Forty ng and 12 ng of MOs were injected into the marginal zone of both blastomeres at the two-cell stage in (**a**) *X. laevis* and (**b**) *X. tropicalis* embryos, respectively. (**a**) Insulin3 MO induced anterior defects in *X. laevis* embryos. Insulin3 MO specifically inhibited the translation of *5′UTR-insulin3-HA*, which has the targeted sequence of MO, but not *mis-insulin3-HA*, which has 7 mismatches in the targeted sequences. (**b**) Xt. insulin3 S MO induced anterior defects in *X. tropicalis* embryos. Xt. insulin3 S MO inhibited normal splicing of *insulin3* mRNA. Full-length blots and gels are presented in Supplementary figure S7.

**Figure 3:**
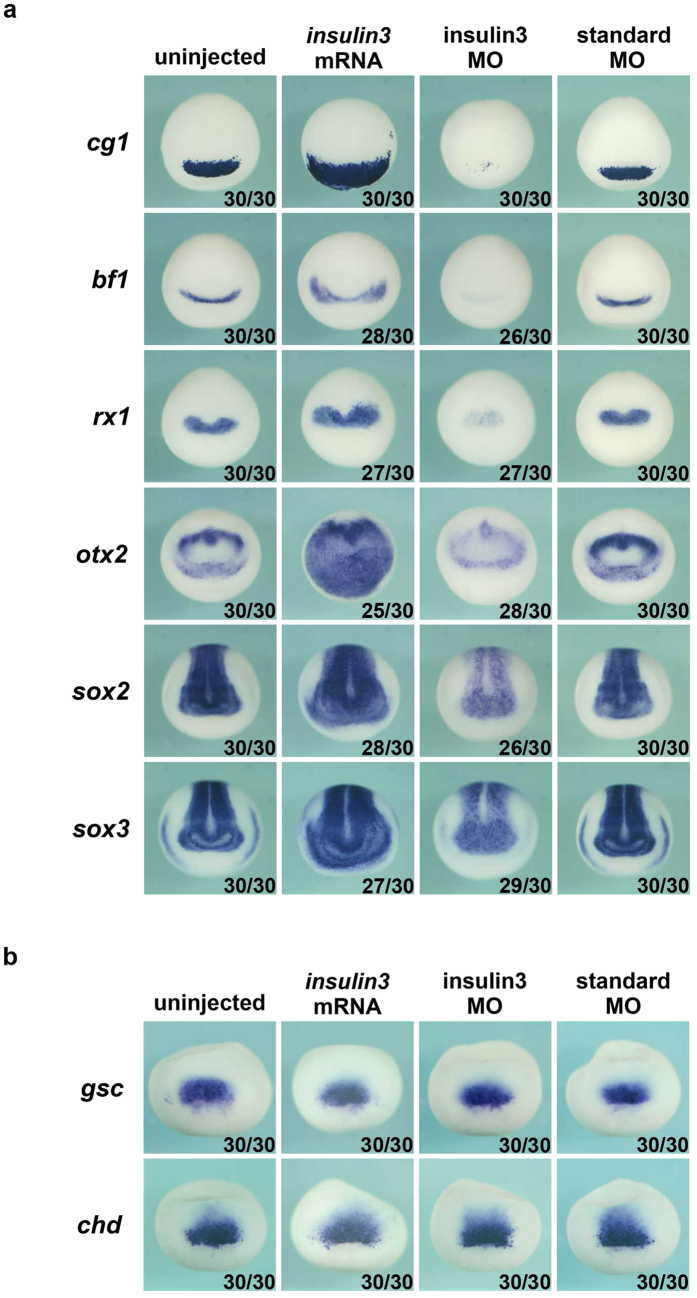
*Insulin3* is essential for anterior neural marker gene expression. *Insulin3* mRNA (300 pg) was injected into the animal pole of both blastomeres at the two-cell stage in *X*. *laevis* embryos. Forty ng of MOs were injected into the marginal zone of both blastomeres at the two-cell stage in *X*. *laevis* embryos. (**a**) Anterior views of stage 15 embryos. Expressions of anterior neural marker genes, *cg1*, *bf1*, *rx1*, *otx2*, *sox2*, and *sox3*, were increased by injection of *insulin3* mRNA. In contrast, insulin3 MO injection decreased the expression of these genes. (**b**) Dorsal views of stage 10.5 embryos. Dorsal mesodermal maker genes, *gsc* and *chd*, were not affected by injection of *insulin3* mRNA or insulin3 MO.

To investigate the role of Insulin3 *in vivo*, we designed translation-inhibiting morpholino oligonucleotides (MO) against the start codon and its surrounding sequences for *X*. *laevis*. Embryos injected with insulin3 MO showed anterior defects with small heads and eyes (Fig. 2a). To confirm this loss-of-function result in *X*. *laevis* embryos, we designed a splice-inhibiting MO for *X*. *tropicalis* (Xt. insulin3 S MO). *X*. *tropicalis* embryos injected with Xt. insulin3 S MO showed the same phenotype with anterior defects (Fig. 2b). Five-mispair control MOs and a standard control MO showed no significant effects (Fig. 2b and Fig. S3). Thus, Insulin3 plays an important role in anterior neural development in both *X*. *laevis* and *X*. *tropicalis* embryos. To examine the function of Insulin3, we injected *insulin3* mRNA and insulin3 MO into the animal pole of each blastomere at the 2-cell stage of *Xenopus laevis* embryos. Five-mispair control MO and a standard control MO showed no effect on the expression of anterior neural maker genes and dorsal mesodermal maker genes (Fig. 2b and Fig. S3b). Injection of *insulin3* mRNA increased cement gland gene, *cg1*, expression and anterior neural gene expression including *bf1*, *rx1*, *otx2*, *sox2*, and *sox3* (Fig. 3a), and injection of insulin3 MO reduced these gene expressions (Fig. 3a), without affecting dorsal mesodermal maker gene expressions, *gsc* and *chd* (Fig. 3b). Pan neural markers, *sox2* and *sox3* were also enhanced in the posterior region of *insulin3* mRNA injected embryos and reduced in the posterior region of insulin3 MO injected embryos. These data indicate that Insulin3 is a neuralising factor in *Xenopus* embryos.

**Figure 4:**
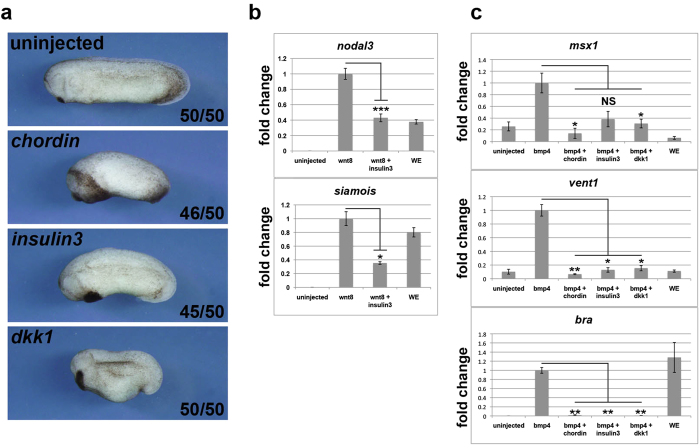
*Insulin3* also inhibit BMP signaling like a Wnt signalling inhibitor, *dkk1*. mRNAs were injected into the animal pole of both blastomeres at the two-cell stage in *X*. *laevis* embryos. (**a**) Amounts of mRNA injected per embryos were: *chd* (25 pg), *insulin3* (250 pg), and *dkk1* (250 pg). Over-expression of *insulin3* shows similar anteriorised phenotype to a Wnt signalling inhibitor, *dkk1*, not dorsalized phenotype like a BMP signaling inhibitor, *chordin*. (**b**,**c**) qRT-PCR analyses of animal caps (**b**) Amounts of mRNA injected per embryos were: *wnt8* (10 pg) and *insulin3* (500 pg). Animal caps were dissected at stage 9 and harvested at stage 10.5. Insulin3 inhibits the expression of Wnt signal target genes, *siamois* and *nodal3*. WE: whole embryo. RT-: reverse transcriptase minus reaction. (**c**) Amounts of mRNA injected per embryos were: *bmp4, chordin*, *insulin3* and *dkk1* (1 ng). *Insulin3* down-regulates expression of BMP target genes, *msx1*, *vent1*, and *bra*, like *dkk1* at St. 10.5 animal caps. Expression level were normalized to *odc*, and scaled to the average value of the wnt8 or bmp4 injected samples; the fold difference between the control and each sample is shown. The data represent mean ± s.e.m (n = 3). *P <0.05; **P < 0.01; ***P < 0.001; (t-test, two tailed).

**Figure 5:**
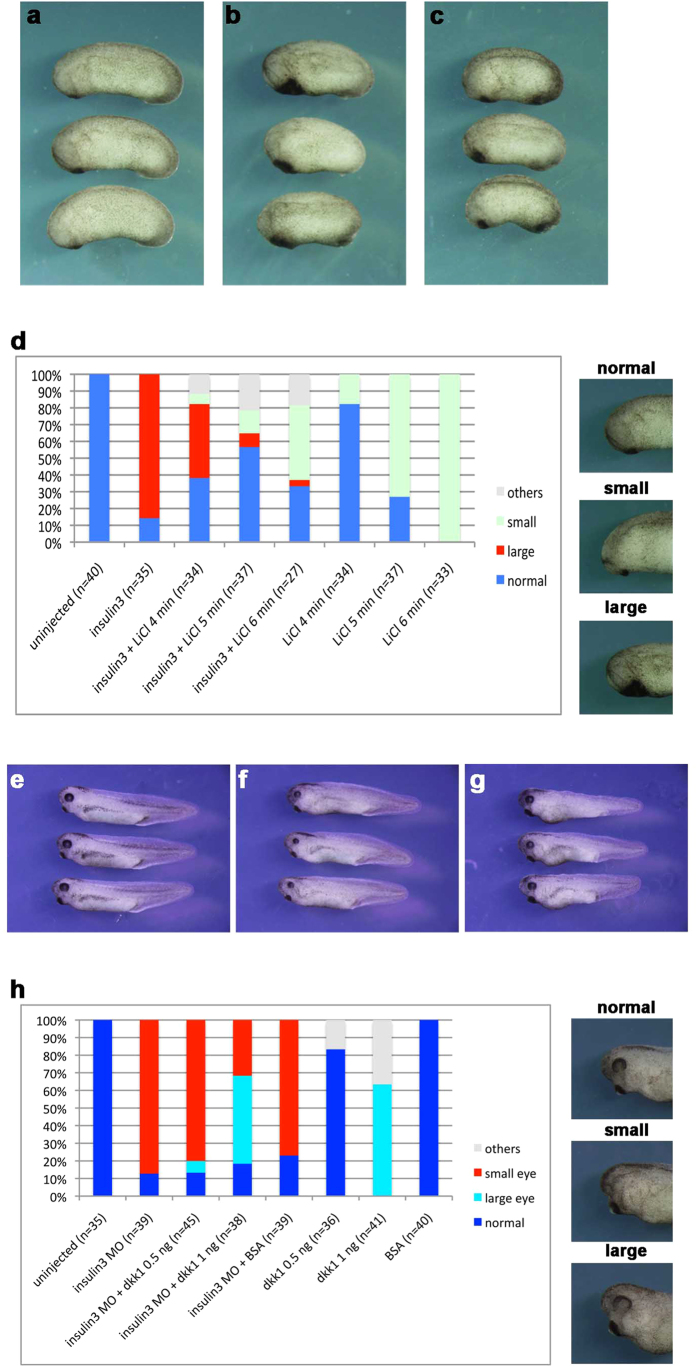
Rescue experiments of *insulin3* over-expressed or knockdown phenotypes by modulation of Wnt signalling. (**a**–**d**) Rescue of *insulin3* over-expressed phenotype by treatment of Wnt signal activator, LiCl. (**a**) Uninjected control embryos. (**b**,**c**) Microinjection of 500 pg of insulin3 mRNA at the two-cell stage induces giant cement gland (**b**). This phenotype is inhibited by treatment of 0.3M LiCl at the gastrulae stage for 5 min. (**c**). (**d**) LiCl treatment can rescue enlarged cement gland induced by insulin3 over-expression. Phenotypic index: Small, small-sized cement gland and head structure. Large, enlarged cement gland. Phenotypes were counted at St. 25. (**e–h**) Rescue of insulin3 MO phenotype by mouse dkk1 protein. (**e**) Uninjected control embryos. (**f**,**g**) Microinjection of 30 ng insulin3 MO at the two-cell stage induces anterior defects with small eyes (**f**). This phenotype is rescued by the injection of mouse dkk1 protein (1 ng) into blastocoel at St. 9 (**g**). (**h**) Dkk1 protein injection into the blastocoel can rescue small-sized eye phenotypes in *insulin3* morphant. Phenotypic index: small, small-sized eye. Large, enlarged eye.

Wnt and BMP inhibition is important to induce anterior neural tissues. To address a question that insulin3 act as a Wnt inhibitor or BMP inhibitor, we verified these activities. *Insulin3* over-expressed embryos are more similar to *dkk1*, a Wnt inhibitor, over-expressed embryos than *chd*, a BMP inhibitor, over-expressed embryos (Fig. 4a). *Insulin3* can inhibit Wnt signalling (Fig. 4b). *Insulin3* and *dkk1* also can inhibit BMP signalling but their activities are obviously weak compared with *chordin* (Fig. 4c). Previous study showed that neural induction in *Xenopus* requires inhibition of Wnt/beta-catenin signalling^21^ and BMP signalling is sensitive to Wnt inhibition in animal caps of early gastrulae embryos^22^. These reports and our results indicate that BMP signal inhibition by insulin3 and dkk1 is the result of Wnt inhibition. Indeed, *insulin3* over-expressed phenotype was rescued by treatment of LiCl, an activator of Wnt signalling at the gastrulae stage, (Fig. 5a–d) and *insulin3* knockdown phenotype by MO was rescued by treatment of mouse dkk1 protein, an inhibitor of Wnt signalling (Fig. 5e–h). These results suggest that Insulin3 mainly acts as a Wnt modulator *in vivo*.

**Figure 6:**
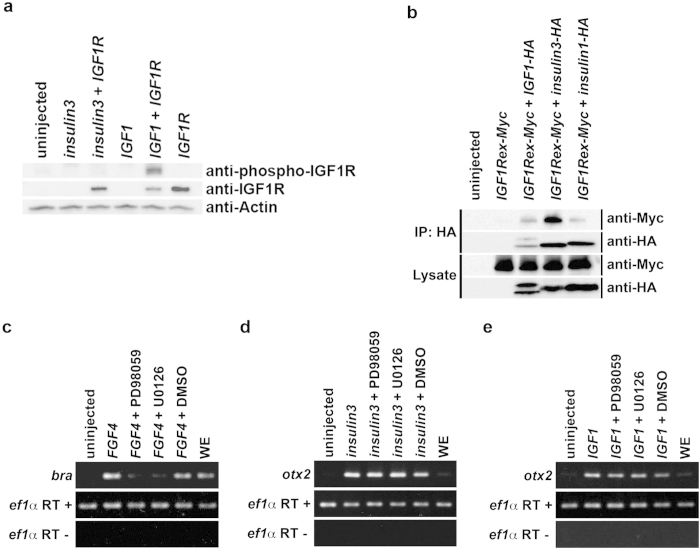
Insulin3 inhibits Wnt signalling but cannot activate the IGF1 receptor. mRNAs were injected into the animal pole of both blastomeres at the two-cell stage in *X*. *laevis* embryos. (**a**) One ng of *insulin3*, *IGF1*, or *IGF1R* mRNA was injected per embryo. Embryos were harvested at stage 11 for western blot analysis. (**b**) One ng of *Myc-tagged extracellular domain of IGF1 receptor* (*IGF1Rex-Myc*), *IGF1-HA*, *insulin3-HA*, or *insulin1-HA* mRNA was injected per embryo. Embryos were harvested at stage 10.5 for co-immunoprecipitation assay. (**c**,**d**,**e**) Amounts of mRNA injected per embryos were: (**c**) *FGF4* (10 pg), (**d**) *insulin3* (100 pg), and (e) *IGF1* (100 pg). Animal caps were dissected at stage 9 and treated with chemical MEK inhibitors, PD98059 (100 μM) and U0126 (100 μM) until sibling embryos reached stage 10.5. (**c**) *Bra* expression induced by *FGF4* was inhibited, but *otx2* expression induced by (**d**) *insulin3* or (**e**) *IGF1* was not. Full-length blots and gels are presented in Supplementary figure S8.

Previous studies reported that IGFs inhibit canonical Wnt signalling^6^,^7^. Here we showed that Insulin3 could also inhibit canonical Wnt signalling (Fig. 4b) indicating it can activate the same signal pathway as IGFs. IGF signals are mainly mediated by means of the IGF1 receptor (IGF1R) and to a lesser extent by the insulin receptor (IR), which are receptor tyrosine kinases (RTKs). We hypothesised that Insulin3 could also activate the IGF1 receptor to induce anterior neural gene expression. Contrary to our hypothesis, our results indicated that Insulin3 cannot activate IGF1R (Fig. 6a), whereas Insulin3 efficiently interacted with the extracellular domain of IGF1R (Fig. 6b). It has been reported that IGFs induce head formation through the IGF/IGF1R/MAPK pathway^5^. Our results indicate that Insulin3 inhibits Wnt signalling using another mechanism.

Next, we attempted to confirm that Insulin3 activity is not mediated through the IGF/IGF1R/MAPK pathway. MEK inhibitors, PD98059 and U0126, efficiently inhibit the expression of *brachyury* (*bra*) induced by FGF4 (Fig. 6c). However, *otx2* expression induced by Insulin3 and IGF1 was not disturbed by PD98059 and U0126 treatment (Fig. 6d,e). This result suggests that the head-enlarging activities of both Insulin3 and IGF1 are not mediated through the IGF/IGF1R/MAPK pathway. We next examined whether IGF1R has synergistic effects on head-enlarging activities with Insulin3 and IGF1. IGF1R and Insulin3 showed no synergistic effects, but, contrary to our expectation, co-injection of *IGF1R* and *IGF1* or *insulin1* mRNAs showed a posteriorising effect and induced protrusions that are usually observed when the MAPK pathway is activated (Supplementary Fig. S4a). In animal caps, *IGF1* and *insulin3* slightly upregulated total Akt and phospho-Akt, and downregulated phospho-Erk. The effect of *insulin3* on Akt was inhibited by co-injection of dominant-negative *IGF1R* (Supplementary Fig. S4b). It has been reported that the Akt pathway positively regulates Wnt signalling^9^. These results indicate that Insulin3 has a novel mechanism for inducing anterior neural genes and imply that IGFs may also activate this novel pathway.

**Figure 7:**
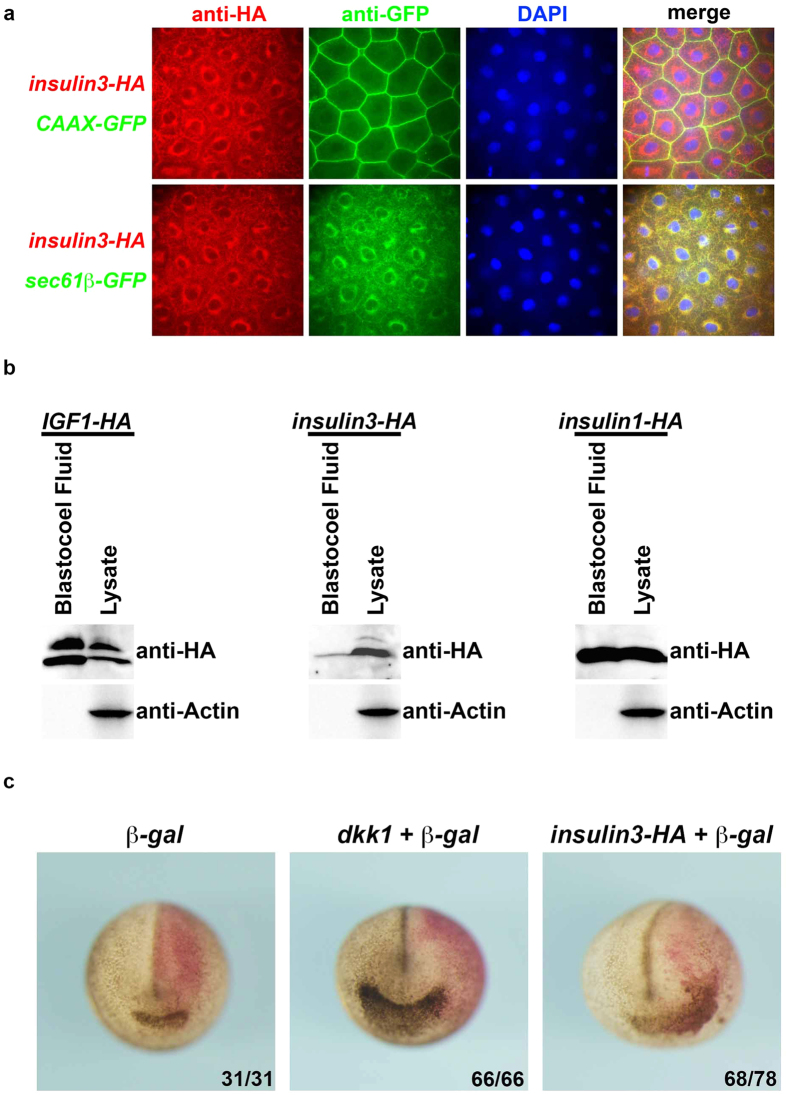
Insulin3 is predominantly localised in the endoplasmic reticulum. (**a**) mRNAs were injected into the animal pole of both blastomeres at the two-cell stage in *X*. *laevis* embryos. Amounts of mRNA injected per embryos were: *insulin3-HA* (500 pg), *CAAX-GFP* (250 pg), and *sec61β-GFP* (250 pg). CAAX-GFP and Sec61β-GFP are localised at the plasma membrane and endoplasmic reticulum, respectively. Embryos were fixed at stage 11. Immunohistochemistry was performed using antibodies against HA-tag and GFP. Animal cap regions were used for analysis. Insulin3-HA was predominantly co-localised with Sec61β-GFP at the endoplasmic reticulum. (**b**) One ng of mRNAs was injected into the animal pole of both blastomeres at the two-cell stage in *X*. *laevis* embryos. Blastocoel fluids were collected at stage 10.5. One μL of the blastocoel fluid was used for western blotting. The lysate was equivalent to one embryo. Insulin3-HA was secreted into the blastocoel at lower efficiency than other insulin family members. Full-length blots are presented in Supplementary figure S9. (**c**) mRNAs were injected into the animal pole of one blastomere at the two-cell stage in *X*. *laevis* embryos. Amounts of mRNA injected per embryos were: *b-gal* (250 pg), *dkk1* (500 pg), and *insulin3-HA* (500 pg). Anterior view of stage 20 embryos. Red-gal staining indicated the injected-side. The effect of *insulin3-HA* was in a cell-autonomous manner, unlike *dkk1*.

To identify the properties of Insulin3 protein, we investigated the cell localisation of HA-tagged Insulin3. Insulin3-HA was secreted at low levels into the extracellular space and was predominantly localised at the ER (Fig. 7a). Western blot analysis using blastocoel fluid also showed that Insulin3-HA was secreted into the blastocoel with low efficiency (Fig. 7b). *Insulin3* activity on anterior region is in a cell-autonomous manner, unlike *dkk1*, which is a secreted inhibitor of Wnt signals (Fig. 7c).

**Figure 8:**
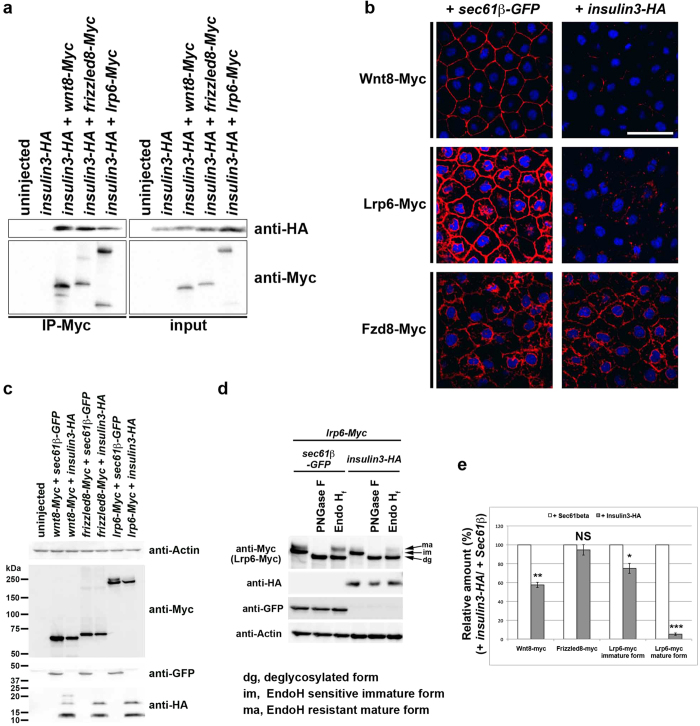
Insulin3 reduced the total amount of extra-cellular Wnt8 and membrane-localised Lrp6, but not Frizzled8. (**a**) Anti-Myc immunoprecipitation (IP). mRNAs was injected into the animal pole of both blastomeres at the two-cell stage in *X*. *laevis* embryos. One ng of *insulin3-HA*, *wnt8-Myc*, *frizzled8-Myc*, or *lrp6-Myc* mRNA was injected per embryo. Embryos were harvested at stage 10.5 for co-immunoprecipitation assay. (**b**,**c**,**d**) mRNAs were injected into the animal pole of both blastomeres at the two-cell stage in *X*. *laevis* embryos. Amounts of mRNA injected per embryos were: *wnt8-Myc* (500 pg), *lrp6-Myc* (500 pg), *frizzled8-Myc* (500 pg), *sec61β-GFP* (1 ng), and *insulin3-HA* (1 ng). *Sec61β-GFP* was used as an ER-localised control instead of cytoplasmic GFP because *insulin3-HA* was localised in ER (Fig. 7). (**b**) Confocal micrographs of ectodermal explants. Embryos were fixed and immunostained with anti-Myc antibody (red) for Wnt8-Myc, Lrp6-Myc, and Frizzled8-Myc at stage 11. Ectodermal explants were dissected after staining and mounted with DAPI. Insulin3 decreased cell-surface levels of Wnt8-Myc, Lrp6-Myc, but not Frizzled8-Myc. (**c**) Western blot analysis of embryo lysates. Insulin3 decreased the total amount of Wnt8-Myc. Insulin3 also reduced the mature form of Lrp6-Myc (upper band) which localised at the plasma membrane. Frizzled8-Myc had no effects. (**d**) Western blot analysis of embryo lysates treated with PNGaseF or EndoH as indicated. Insulin3 reduced EndoH-resistant mature form of Lrp6. dg, deglycosylated form; im, EndoH-sensitive immature form; ma, EndoH-resistant mature form. (**e**) Quantification of the Western blotting in (**c**) and (**d**). The protein bands in the blots from three independent experiments were quantified by using ImageJ for densitometry. The amount of protein in control (+Sec61β) was designated as 100%. Error bar indicate the SEM of these experiments. The data are presented as mean ± s.e.m. NS (not significant, *P* > 0.05), *P < 0.05; **P < 0.01; ***P < 0.001; (*t*-test, two tailed). Full-length blots are presented in Supplementary figure S9.

To determine the molecular mechanism by which Insulin3 inhibits Wnt signalling, we performed co-immunoprecipitation and immunohistochemistry assays. Insulin3 can interact with Wnt components, Wnt8, Frizzled8 and Lrp6 (Fig. 8a). Co-expression of Insulin3-HA inhibited extracellular localisation of Wnt8-Myc and Lrp6-Myc, but not Fzd8-Myc (Fig. 8b). Co-injection of *insulin3-HA* reduced the total amount of Wnt8-Myc and Lrp6-Myc, but not Frizzeled8-Myc (Fig. 8c,d). Lrp6-Myc was detected as upper and lower bands, which might correspond to the mature cell surface localised form (ma) and immature cytoplasmic form (im) of Lrp6, respectively (Fig. 8c)^20^. Indeed, the upper band of Lrp6-Myc was resistant to Endoglycosidase H (EndoH), which cleaves immature N-glycans with high mannose (Fig. 8d). Co-injection of *insulin3-HA* with *lrp6-Myc* especially reduced the upper band of Lrp6-Myc. Thus, Insulin3-HA especially reduced the mature form of Lrp6 and inhibited its localisation on the cell surface (Fig. 8b,e).

## Discussion

Here we report the isolation of a novel insulin-like factor, *insulin3*, which attenuated canonical Wnt signalling. *Insulin3* can inhibit target gene expressions of Wnt/β-catenin signalling (Fig. 4b). Over-expression of *insulin3* induces expansion of anterior structure with giant cement gland. This phenotype was rescued by treatment of LiCl, an activator of Wnt signalling at the gastrulae stage (Fig. 5a–d). Loss-of-function analysis by insulin3 MO injection demonstrated a predominant defect in anterior neural development and this phenotype was rescued by treatment of mouse dkk1 protein, an inhibitor of Wnt signalling (Fig. 5e–h). These results indicate that Insulin3 functions as a Wnt inhibitor. Previous report showed that inhibition of Wnt-β-catenin signalling is required for neural induction in *Xenopus*^21^. Pan neural markers, *sox2* and *sox3* were upregulated in the *insulin3* mRNA injected embryos and down-regulated in the insulin3 MO injected embryos (Fig. 3a). These data indicate that Insulin3 is a neuralising factor in *Xenopus* embryos.

Insulin3 has a putative signal peptide for secretion. However, Insulin3 is not efficiently secreted into the extracellular space and is predominantly localised in the ER when an anterior neural marker *otx2* is already induced by *insulin3* over-expression (Fig. 7a,b). We tried to elucidate the role of insulin3 in ER. However, we cannot exclude the possible function of insulin3 in extracellular space. It is still uncertain whether insulin3 functions in ER or extracellular space. Further investigation is required for this matter. Insulin3 could not activate IGF1R and Insulin3 did not show synergistic effect with IGF1R to induce anterior structures (Fig. 6a and Supplementary Fig. S4). These results indicate that Insulin3 induces anterior neural genes in an IGF1R-independent manner. In this report, we identified two novel mechanisms to inhibit Wnt signals by the reduction of mature-glycosylated Lrp6 and degradation of Wnt ligands in a cell-autonomous manner. Insulin3 reduced Lrp6 at the plasma membrane and the total amount of Wnt ligands in the extracellular space, resulting in the retardation of Wnt signalling. *Lrp6* are expressed maternally and ubiquitously through early development^23^. *Wnt3A* and *wnt8* are expressed in the neural plate of early neurulae^24^,^25^. At least, these factors will be a candidate for direct target of Insulin3 *in vivo*.

IGF signalling is mediated by activating the two main intracellular pathways: the MAPK pathway and PI3 kinase pathway. A previous report clearly showed that the expression of *otx2* induced by IGF was not affected by *Δp85*, a dominant negative regulatory subunit of PI3K, a dominant negative Akt (dnAkt) or LY294002, a chemical PI3K inhibitor^2^. Another report showed that PI3K activation by p110α, a constitutively active form of the PI3K catalytic subunit, inhibited GSK3β resulting in activating Wnt signals^9^. Taken together, it suggests the IGF/IGF1R/PI3K pathway cannot contribute to Wnt signal inhibition by IGF ligands. The IGF/IGF1R/MAPK pathway is a possible candidate for Wnt signal inhibition. In particular, IGF/IGF1R/MAPK signalling has been implicated in neural induction^5^. However, our results did not support this. *Otx2* expression induced by *IGF1* mRNA injection was not disturbed by treatment with MEK inhibitors, PD98059 and U0126 (Fig. 6e). Co-injection of *IGF1* and *IGF1R* mRNAs did not show synergetic effects on anterior expansion in *Xenopus* embryos (Supplementary Fig. S4). On the contrary, co-injection of *IGF1* and *IGF1R* mRNAs showed posteriorising effects that are usually observed when FGF/MAPK signals are activated (Supplementary Fig. S4).

When comparing the activity of Insulin3 with IGF1, we found that *IGF1* mRNA injection induced a giant cement gland, but IGF1 protein treatment did not (Supplementary Fig. S5a). Injection of Dkk1 protein, a Wnt inhibitor, into the blastocoel can induce a giant cement gland similar to *dkk1* mRNA injection. FGF4 protein injection caused a posteriorising effect similar to *FGF4* mRNA injection. Lefty protein injection showed a significant gastrulation defect usually observed when nodal signalling is inhibited by *lefty* mRNA injection (Supplementary Fig. S5a). IGF1 protein can activate the IGF1R downstream targets Akt and Erk in *X*. *laevis* oocytes (Supplementary Fig. S5b). However, IGF1 protein injection showed no effect on early *Xenopus* development, indicating extracellular IGF1 do not contribute to the anterior neural induction. Our results indicate that the IGF/IGF1R pathway acts as a posteriorising signal during the early developmental stages and does not contribute to the induction of anterior neural tissues. The major mediators of IGF1R/IR signalling are the insulin receptor substrates (IRS). Loss of IRS1 function in *Xenopus* embryos affects the expression of the eye marker genes *rx1* and *pax6*, but not the pan-neural marker gene *sox3*^26^. These results support our idea that canonical IGF1R signalling does not contribute to the induction of anterior neural tissues.

IGF1 also inhibited Wnt signalling in early *Xenopus* embryos^6^,^7^. We attempted to identify whether IGF1 had the same effects as Insulin3. However, IGF1 effects on Wnt degradation and Lrp6 maturation were slightly different from Insulin3. IGF1-HA degraded Wnt3a-Myc but not Wnt8-Myc (Supplementary Fig. S6a,b). A previous report showed that *wnt3a* expression is strongly inhibited in *FAK* morphants with a giant cement gland, while *wnt8* expression is normal, indicating that *wnt3a* inhibition is sufficient to anteriorise *Xenopus* embryos^15^. Consistent with this observation, we showed that IGF1 degraded Wnt3a but not Wnt8, and could anteriorise *Xenopus* embryos. IGF1-HA had a slight effect on the mature form of Lrp6 and increased the non-glycosylated form of Lrp6 (lower band) (Supplementary Fig. S6c). Thus far, it has been suggested that diverse activities of IGFs might be mediated through IGF1R and IR. Our results explained the IGF1R-independent novel mechanisms of Wnt inhibition that contribute to the significant bioactivities of insulin-like factors. These results indicate that IGF1 has similar activities to Insulin3 but there are some differences in activity between insulin-like ligands. Furthermore, these differences suggest that its mechanism is more complex and that important systems involving unidentified factors may also exist in addition to these. We do not deny the possibility that IGFs are activating unknown signals. Further research is required to elucidate the whole mechanism.

## Methods

### Approvals of animal experiment

The protocols for the use of animals in this study were approved by Office for Life Science Research Ethics and Safety of the University of Tokyo (Project No.19-2, 23-11) and all experiments were carried out in accordance with the approved protocols.

### Plasmid Constructs, Morpholino Oligonucleotides and mRNAs

*Insulin3* [Contig029272 released from XDB3 (http://xenopus.nibb.ac.jp/)] was identified through a Blast search of the *Xenopus* EST database using *X. laevis insulin* sequence (ins-a: NM_001085882). pGEM-insulin3 was cloned by RT-PCR amplified from *X. laevis* stage 10 cDNA using primers (forward, GTGACTTATTGGAATGGGTTG; reverse, AGGCTAATAACATGCCAG). pCS2p-insulin3 was cloned by PCR amplification from pGEM-insulin3 using primers (forward, CACAGAATTCATGGCACAAGGTGATTGGGC; reverse, CACACTCGAGTTATCGATTACAGTAATGCTCC) with engineered *Eco*RI and *Xho*I sites and cloned directionally into pCS2p+. pCS2p-insulin3-HA has an HA-tag sequence at the C-terminus. pCS2p-5′UTR-insulin3-HA has MO targeted sequences, GAAT**ATG**GCACAAGGTGATTGGGCA. pCS2p-mis-insulin3-HA has a mutated sequence, ATTC**ATG**GCTCAGGGAGACTGGGCT, at the MO targeted site. Bold letters indicate the start codon. Underlined letters indicate silent mutations. To generate a *Myc-tagged extracellular domain of IGF1R* (pCS2-IGF1Rex-Myc), 6 Myc tags were connected at the C-terminus of a DN-IGF1R construct^6^. pCS2-IGF1-HA has an HA-tag (YPYDVPDYA) between 118A and 119R, just after the C-terminus of mature IGF1 protein. pCS2p-insulin1 has an ORF sequence from *X. laevis*. pCS2p-insulin1-HA has an HA-tag at the C-terminus. pCS2-hlrp6-Myc was generated using pCS2-hlrp6^27^ as the template and has 6 Myc tags at the C-terminus. pCS2p-FGF4 has an ORF sequence from *X*. *tropicalis FGF4*. pCS2-frizzled8-Myc has an ORF sequence from *X*. *tropicalis frizzled8* and 6 Myc tags at the C-terminus. Capped mRNAs were synthesized by *in vitro* transcription of plasmids using SP6 mMessage mMachine kits (Ambion). The expression vectors used for mRNA synthesis were: pCS2p-insulin3, pCS2p-insulin3-HA, pCS2p-5′UTR-insulin3-HA, pCS2p-insulin1, pCS2p-insulin1-HA, pCS2-IGF1-HA, pCS2-IGF1Rex-Myc, pCS2-hlrp6-Myc, pCS2-frizzled8-Myc, pCS2p-FGF4, pCS2-plasma membrane-localised GFP (CAAX-GFP)^28^, pCS2-IGF1^6^, pCS2-IGF1R^6^, pCS2-dominant-negative IGF1R (DN-IGF1R)^6^, pCS2-sec61β-GFP^29^, pCSf107mT-wnt8-myc^30^, pSP64T-wnt8^31^,^32^,^33^, and pCS2-NLS-lacZ^34^. A translation-targeted antisense morpholino oligonucleotide against *X*. *laevis insulin3*, TGCCCAATCACCTTGTGCCATATTC (insulin3 MO), was designed at the translational start region (the underlined sequence corresponds to the start codon). Based on the genome sequence released from JGI, a splicing-targeted antisense morpholino oligo nucleotide against *X*. *tropicalis insulin3*, TGCAAGAAATGAAAGTTACCTTTTT (Xt. insulin3 S MO) was designed at the exon-intron boundary. Underlined letters correspond to the exon region. The 5-bp mismatch morpholinos used were TaCCaAATaACCTTaTGCaATATTC (insulin3 5mis MO) and TaCAAaAAATaAAAaTTACaTTTTT (Xt. insulin3 S 5mis MO) (the substituted bases are lowercase). The sequence of the Gene Tools control morpholino was CCTCTTACCTCAGTTACAATTTATA.

### Embryo Manipulation and Microinjection

*In vitro* fertilization and microinjection of *X*. *laevis* and *X*. *tropicalis* embryos were performed as previously described^35^. Embryonic stages were determined as defined as previously described^36^. For the animal cap assay, synthesized RNA was microinjected into the animal side of both blastomeres of 2-cell stage embryos. Animal caps were dissected at stage 9 and were cultured in Steinberg’s solution (SS) until stage 10.5 or 11 for RT-PCR. PD98059 (Calbiochem) and U0126 (Calbiochem) were dissolved in DMSO and stored at a 10 mM concentration. Animal caps were treated with up to 100 μM PD98059 or U0126. LiCl treatment was performed at the gastrulae stage for 4–6 min in 0.3 M LiCl and subsequent washing.

### *In situ* hybridization analysis

*In situ* hybridization analysis was performed as previously described^37^. DIG-labelled antisense RNA probes were synthesized with SP6, T7, or T3 polymerase (Promega) using the following plasmids: pGEM-insulin3, pBluescriptSK(−)-goosecoid^38^, pBluescript SK(−)-chordin^13^, pXCG-1^39^, pCS2-bf1^40^, pGEM-rx1^41^, pBluescriptSK(−)-otx2^42^, pGEM-sox2^43^, and pBluescriptKS(+)-sox3^43^.

### RT-PCR

Total RNA isolation and RT-PCR methods were described previously^44^. The primer pairs for *bra*, *noggin*, *follistatin*, *otx2*, and *odc* are described in Xenbase (http://www.xenbase.org/other/static/methods/RT-PCR.jsp). Other primers used were: *chd*^13^, *cerberus*^10^, *shisa*^45^ *sox3*^46^, *ef1*α (for *X*. *laevis*)^47^, *ef1*α (for *X*. *tropicalis*)^48^, *insulin*^49^, *insulin3* (*X*. *laevis*) (forward 5′-ATGGCACAAGGTGATTGGGC-3′ and reverse 5′-CGGTAACAGCACTTTTCCACTATGC-3′), *insulin3* (*X*. *tropicalis*)(forward 5′-TCTTGGTGTGTGAAGGCAGAGG-3′ and reverse 5′-CGGTAACAGCACTTTTCCACTATGC-3′). *Odc* and *ef1*α were used as internal controls. Reverse transcriptase negative (RT-) reactions were included to indicate the absence of genomic DNA contamination.

### Quantitative RT–PCR

Total RNA of either three whole embryos or 20 animal caps per sample was extracted using the ISOGEN (Nippongene), and cDNA synthesis was carried out using oligo dT (Roche) and SuperScript® III Reverse Transcriptase (Life Technologies). Quantitative RT–PCR was performed with a CFX96 real-time PCR detection system (BIO-RAD) using THUNDERBIRD^®^ SYBR^®^ qPCR Mix (TOYOBO). Measurements were performed in triplicates and normalized to the expression levels of *odc*. The sequences of the primer pairs were previously described: *odc*^50^, *siamois*^50^, *nodal3*^50^, *bra*^50^, *vent1*^51^, and *msx1*^52^.

### Western blotting and co-immunoprecipitation assays

Blastocoel fluids were collected as described previously^53^. Embryos and oocytes were homogenized in 10 μL/embryo or oocyte lysis buffer [Dulbecco phosphate buffered saline (PBS) with 1% Triton X-100 and protease inhibitor cocktail (Roche)]. After centrifugation, supernatants were treated with protein G-sepharose (Amersham Bioscience) bound with anti-c-Myc or anti-HA antibodies for co-immunoprecipitation assay. The following antibodies were used for immunoprecipitation: IP, anti-c-Myc (9E10) antibody (Santa Cruz Biotechnology) and anti-HA antibody HA-probe (Y-11) (Santa Cruz Biotechnology). Western blots were carried out under reducing conditions, as previously described^35^. Antibodies used were anti-c-Myc (9E10)-HRP antibody (Santa Cruz Biotechnology), anti-HA-Peroxidase, High affinity (3F10) (Roche), anti-phospho-IGF1 receptor b (Tyr1135/1136) Insulin receptor b (Tyr1150/1151) (19H7) (Cell Signaling), anti-IGF1 receptor antibody (Abcam), anti-phospho-Akt (Ser473) antibody (Cell Signaling), anti-PKBa/Akt antibody (BD Biosciences), anti-MAP Kinase, Activated (Diphosphorylated Erk1/2) Clone MAPK-YT (Sigma), anti-p44/42 MAPK (Erk1/2) antibody, anti-GFP (FL) rabbit polyclonal antibody (Santa Cruz Biotechnology), anti-rabbit IgG HRP linked antibody (Cell Signaling), and anti-mouse IgG HRP linked antibody (Cell Signaling). An anti-Actin antibody (AC-40, Sigma) was used as a loading control. Phosphatase inhibitor cocktail 1 and 2 (Sigma) were used when necessary. For PNGsaseF and EndoHf treatments (New England BioLabs), 9 μL lysate (10 μL/embryo) were used according to the manufacturer’s instructions.

### Protein injection

Recombinant human IGF1 (Cell Signaling), recombinant mouse Lefty1, recombinant mouse FGF basic (bFGF) (R&D), and recombinant mouse Dkk1 (R&D), were injected into the blastula cavity. These proteins were dissolved in Steinberg’s solution (SS) with 0.1% BSA (Sigma).

### Oocyte manipulation

Manual defolliculation of oocytes was carried out as described previously^37^. Oocytes were cultured in OR2 medium (0.1% BSA) with 100 ng/mL or 1 μg/mL IGF1 for 3 h.

### Immunofluorescent staining

Immunostaining was carried out as previously described^37^. Primary antibodies included: anti-HA monoclonal antibody (HA.11 Clone 16B12) (Covance), anti-GFP rabbit polyclonal antibody (Abcam), anti-c-Myc (9E10) antibody (Santa Cruz Biotechnology) and were used at 1:500 dilution. Secondary antibodies included: Alexa Fluor (AF) 594 conjugated anti-mouse IgG or anti-rabbit IgG and AF488-conjugated anti-rabbit IgG (Molecular Probes) and were used at 1:200 dilution. To stain *Xenopus* embryos, whole embryos were fixed by MEMFA for 1 hr and explants were mounted in Prolong Gold (Invitrogen). Digital confocal images were captured with Olympus DSU-Olympus IX81 or Olympus FV1000 confocal microscopes.

## Additional Information

**How to cite this article**: Haramoto, Y. *et al.* Insulin-like factor regulates neural induction through an IGF1 receptor-independent mechanism. *Sci. Rep.* **5**, 11603; doi: 10.1038/srep11603 (2015).

## Supplementary Material

Supplementary Information
