# Supplementary material for: Insulin-like factor regulates neural induction through an IGF1 receptor-independent mechanism
Source: Sci Rep. 2015 Jun 26;5:11603. doi: 10.1038/srep11603 (PMC4481404; doi:10.1038/srep11603)
Supplement: Supplementary Information [file srep11603-s1.pdf]

Supplemental Information

Insulin-like factor regulates neural induction through an IGF1 receptor-independent mechanism

Yoshikazu Haramoto[1], Shuji Takahashi[2, 3*], Tomomi Oshima[1†] , Yasuko Onuma[1], Yuzuru Ito[1], Makoto Asashima[1]

[1]Research Center for Stem Cell Engineering, National Institute of Advanced Industrial Science and Technology (AIST), Tsukuba Central 4, 1-1-1 Higashi, Tsukuba, Ibaraki 305-8562, Japan, [2]Institute for Amphibian Biology, Graduate School of Science, Hiroshima University, 1-3-1 Kagamiyama, Higashi-Hiroshima, Hiroshima 739-8526, Japan and [3]Center for Structuring Life Sciences, Graduate School of Arts and Sciences, The University of Tokyo, 3-8-1 Komaba, Meguro-ku, Tokyo 153-8902, Japan. †Present address: Department of Physiology, Kitasato University School of Medicine, 1-15-1 Kitasato, Minami, Sagamihara, Kanagawa, 252-0374, Japan.

*Corresponding author: Shuji Takahashi, Institute for Amphibian Biology, Graduate School of Science, Hiroshima University, 1-3-1 Kagamiyama, Higashi-Hiroshima, Hiroshima, 739-8526, Japan
Tel.: +81-82-424-7125. Fax:+81-82-424-0739, Email: shujit@hiroshima-u.ac.jp

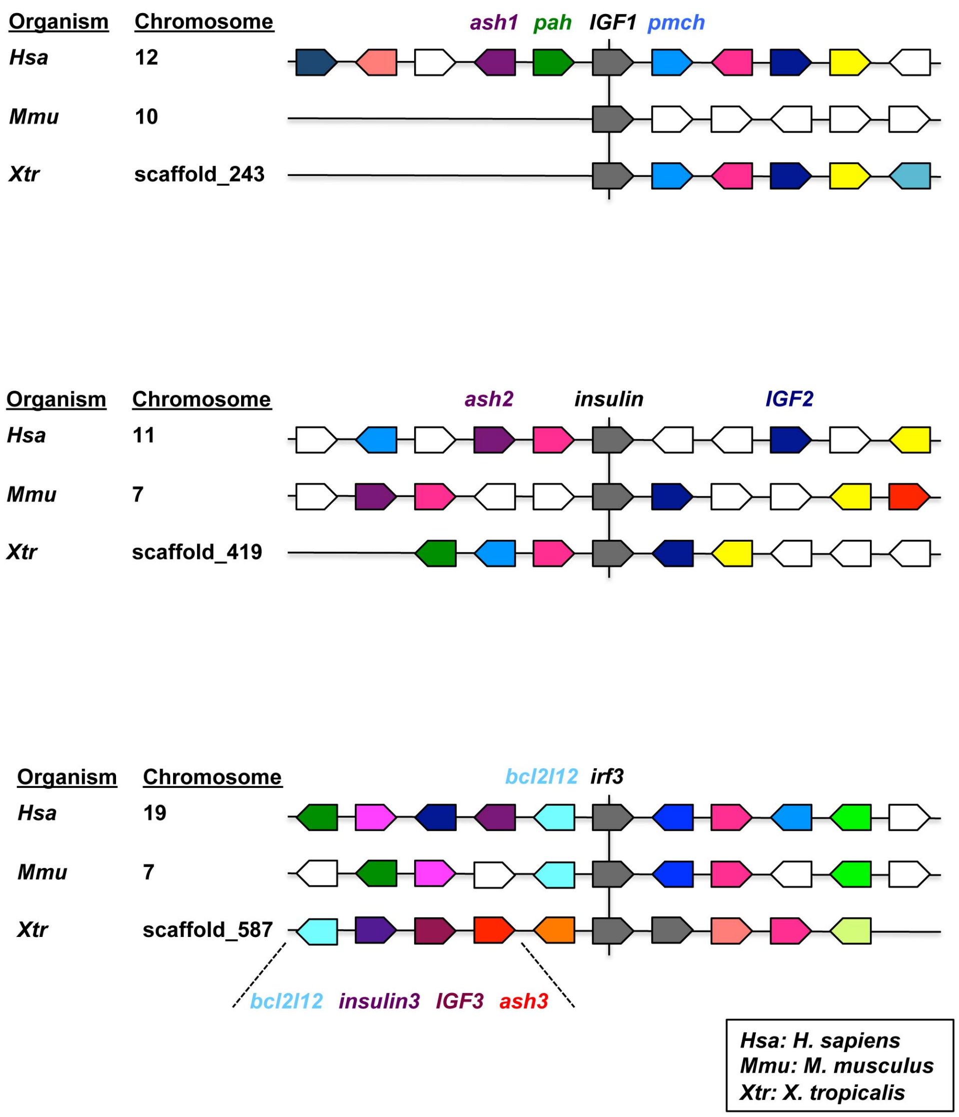

Figure S1. Synteny analysis of *insulin/IGF* locus.
Using Metazome [(http://www.metazome.net/) ©2006-2014 University of California Regents], the flanking upstream and downstream genes of the *Insulin* and *IGF* orthologs were compared between *H. sapiens*, *M. musculus*, and *X. tropicalis*. The gene and the transcriptional direction are indicated by the colour and direction of the box, respectively. The synteny around *IGF1* locus is conserved between *H. sapiens* and *X. tropicalis*. The synteny around *IGF2* locus is conserved among these three species. *IGF2* and *insulin* are located nearby. The locus of the *insulin3* and *IGF3* is not conserved among other species.

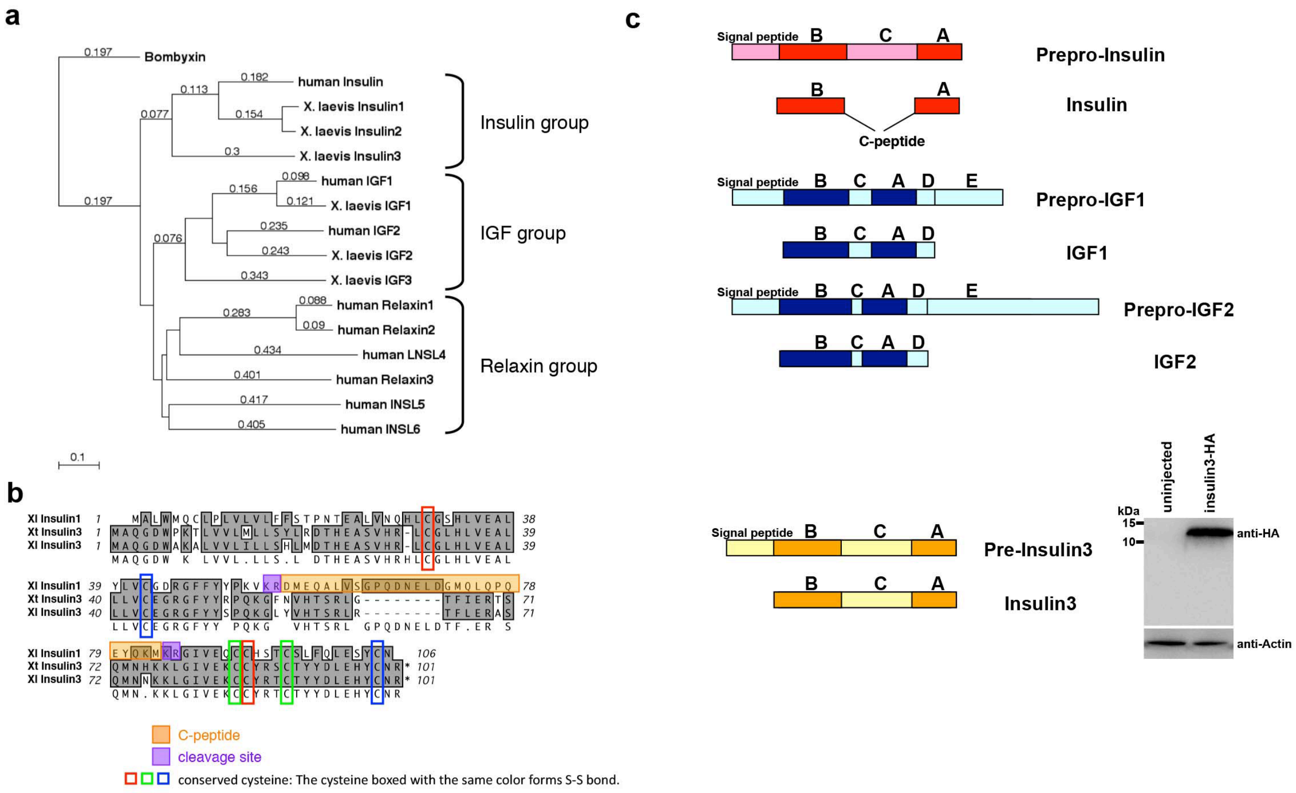

Figure S2. Protein features of Insulin3.

(a) Phylogenetic analysis using protein sequences of the Insulin family. The phylogenetic tree was calculated by MacVector 11.1.0 software. Human Insulin (AAA59172), human IGF1 (CAA01955), human IGF2 (NP_000603), human Relaxin1 (CAA00599), human Relaxin2 (AAI26416), human Relaxin3 (AAI40936), human LNSL4 (AAH26254), human INSL5 (AAQ89389), human INSL6 (AAD39003), *X. laevis* Insulin1 (NP_001079351), *X. laevis* Insulin2 (NP_001079350), *X. laevis* Insulin3 (Translation of Contig029272 released from XDB3), *X. laevis* IGF1 (NP_001156865), *X. laevis* IGF2 (AAL11445), and *X. laevis* IGF3 (NP_001082137) were tested. Silkworm Bombyxin (BAA00246) was used as the outgroup. (b) Amino acid sequence alignment indicates that Insulin3 has 6 cysteine residues conserved among the Insulin family, and no proteolytic cleavage sites that are required to release C-peptide. The cysteine residues boxed with the same colour form intramolecular S-S bands. (c) Schematic illustration of Insulin, IGF1, IGF2, and Insulin3 protein processing. Insulin and IGF molecules are synthesized as inactive prepropeptides that are converted to a mature active form by endoproteolysis, thereby releasing C-peptide and E-peptide, respectively. Insulin3-HA has an HA tag at the C-terminus and shows the same activity as the native form (data not shown). *Insulin3-HA* mRNA (1 ng) was injected into the animal pole of two-cell-stage *X. laevis* embryos. Embryos were harvested at stage 10.5. Western blot analysis showed that Insulin3-HA is not proteolytically processed to release C-peptide.

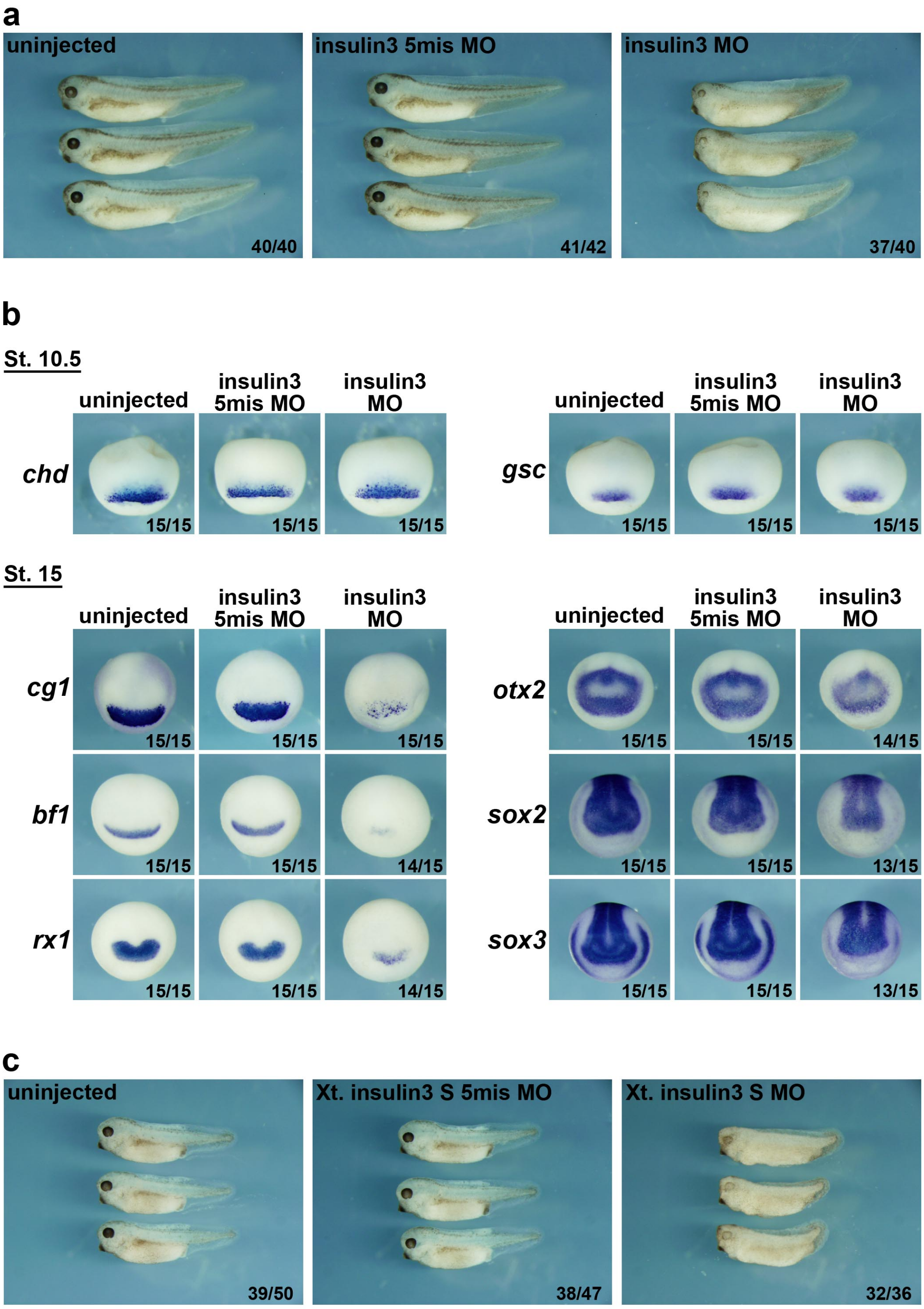

Figure S3. Five-mismatch MOs showed no significant effects.
(a, b) Forty ng of MOs were injected into the marginal zone of both blastomeres at the two-cell stage in *X. laevis* embryos. (a) Insulin3 5mis MO showed no effect on *X. laevis* development, unlike insulin3 MO. (b) Dorsal views of stage 10.5 embryos. Dorsal mesodermal maker genes, *gsc* and *chd*, were not affected by injection of insulin3 5mis MO or insulin3 MO. Anterior views of stage 15 embryos. Expressions of anterior neural marker genes, *cg1*, *bf1*, *rx1*, *otx2*, *sox2*, and *sox3*, were not affected by injection of insulin3 5mis MO, unlike insulin3 MO. (c) Twelve ng of MOs were injected into the marginal zone of both blastomeres at the two-cell stage in *X. tropicalis* embryos. Xt. insulin3 S 5mis MO showed no effects on *X. tropicalis* development, unlike Xt. insulin3 S MO. Numbers of embryos with the shown phenotype are indicated in panels.

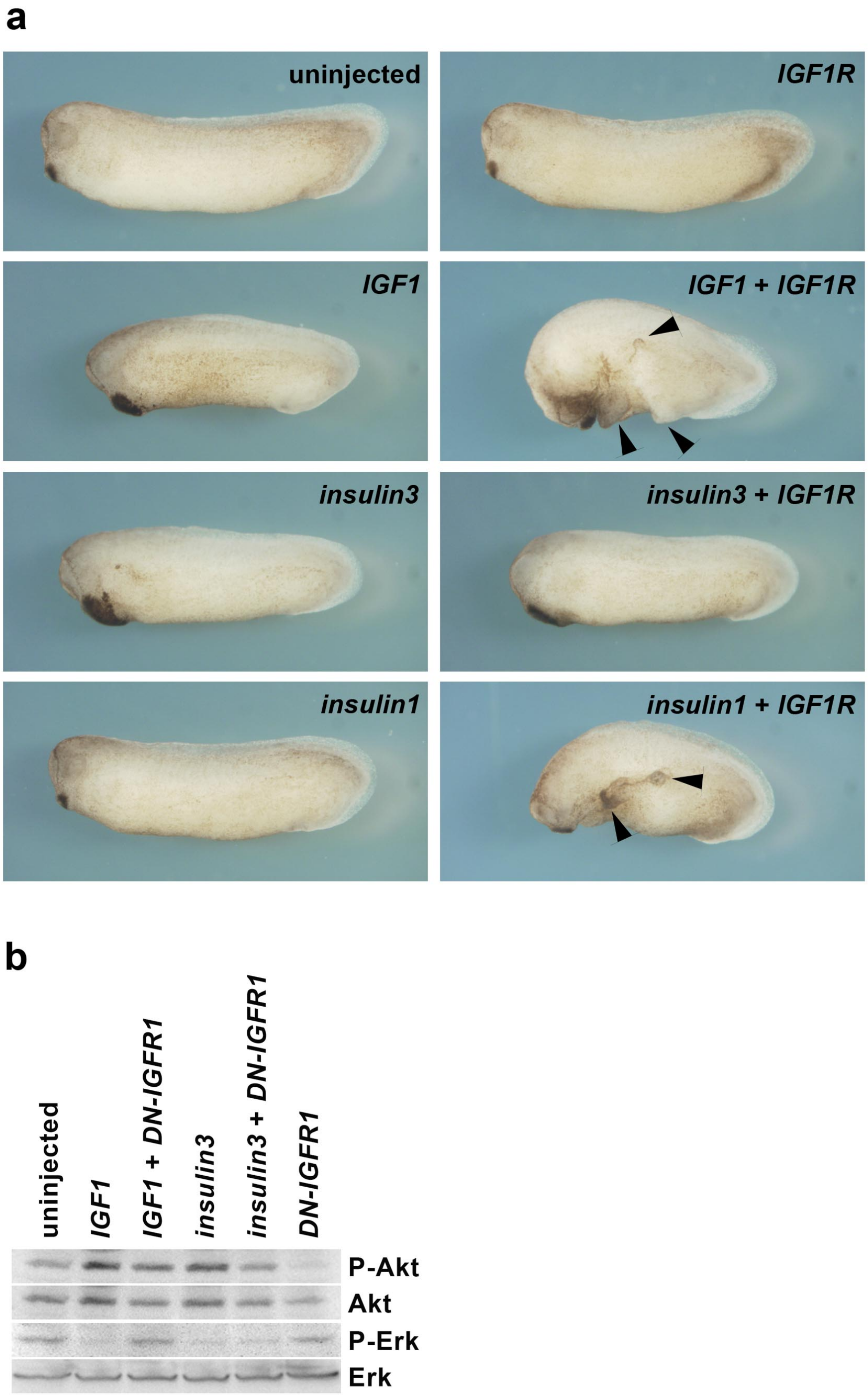

Figure S4. Insulin3 and IGF1R did not show synergistic effects for anteriorisation.
(a) mRNAs were injected into the animal pole of both blastomeres at the two-cell stage in *X. laevis* embryos. Embryos were fixed at stage 30. Amounts of mRNA injected per embryos were: *IGF1* (500 pg), *insulin3* (500 pg), *IGF1R* (1 ng), and *insulin1* (2 ng). Injected RNA is indicated in each panel. Co-injection of *IGF1R* and *IGF1* or *insulin* mRNAs showed a posteriorising effect and induced protrusions (arrowheads). Numbers of embryos with the shown phenotype are *IGF1* (29/30), *IGF1* + *IGF1R* (21/30), *insulin*3 (30/30), *insulin3* + *IGF1R* (28/30), *insulin* (30/30), *insulin* + *IGF1R* (11/30). (b) Western blot analysis of animal caps. mRNAs were injected into the animal pole of both blastomeres at the two-cell stage in *X. laevis* embryos. Amounts of mRNA injected per embryos were: *IGF1* (1 ng), *insulin3* (1 ng), and *DN-IGF1R* (1 ng). Animal caps were dissected at stage 9 and were cultured in Steinberg's solution (SS) until stage 11.

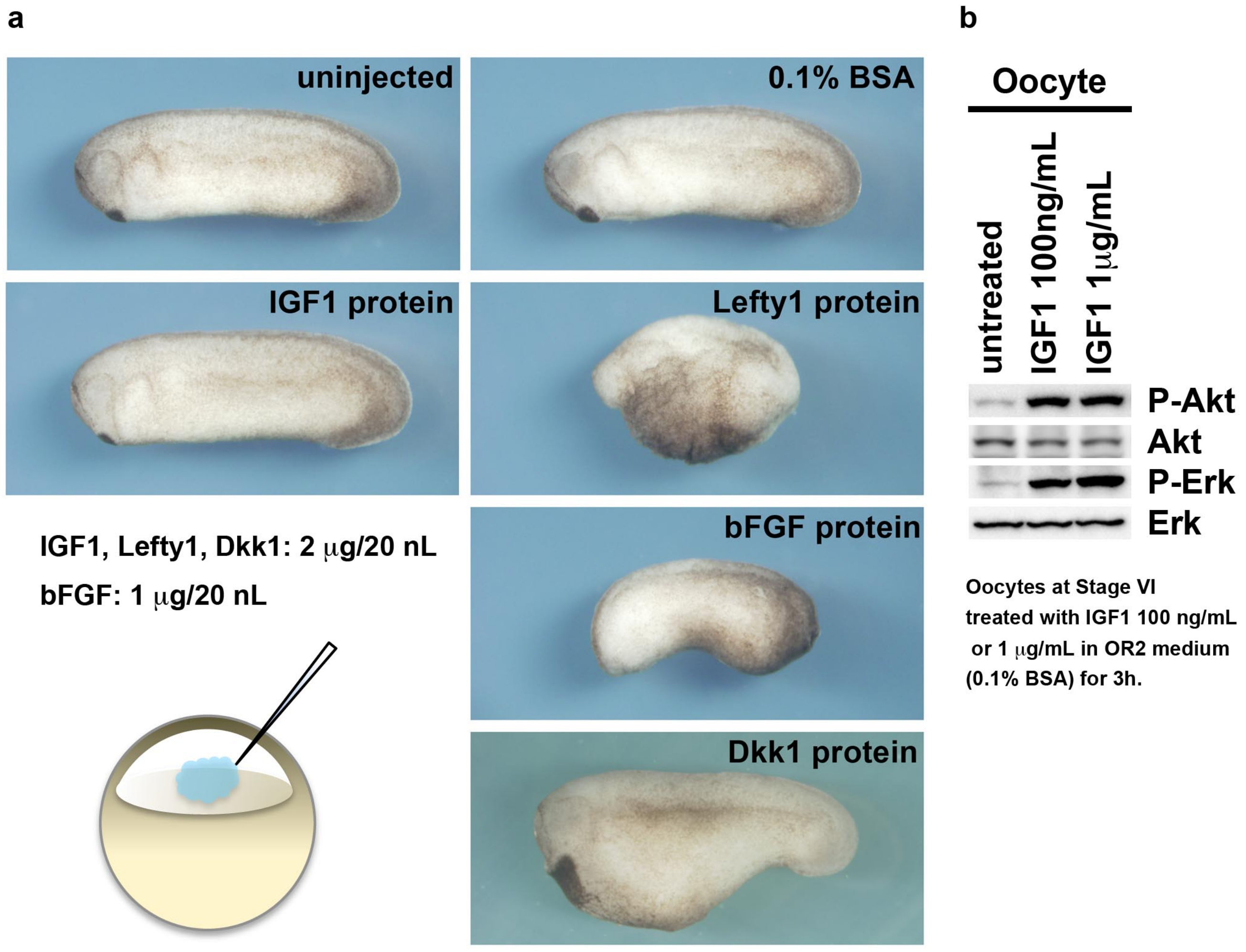

Figure S5. IGF1 protein treatment did not show any effect on anterior development.
(a) Recombinant proteins were injected into the blastocoel. Amounts of protein injected per embryos were: BSA, IGF1, Lefty1, Dkk1 (2 μg/20 nL, respectively), and bFGF (1 μg/20 nL). The ratio of embryos with the shown phenotype were 100% (n≥30). (b) Oocytes at stage VI were treated with recombinant human IGF1 in OR2 medium (0.1% BSA) for 3 h. Recombinant human IGF1 can activate downstream targets of IGF1 receptor, Akt and Erk in oocytes.

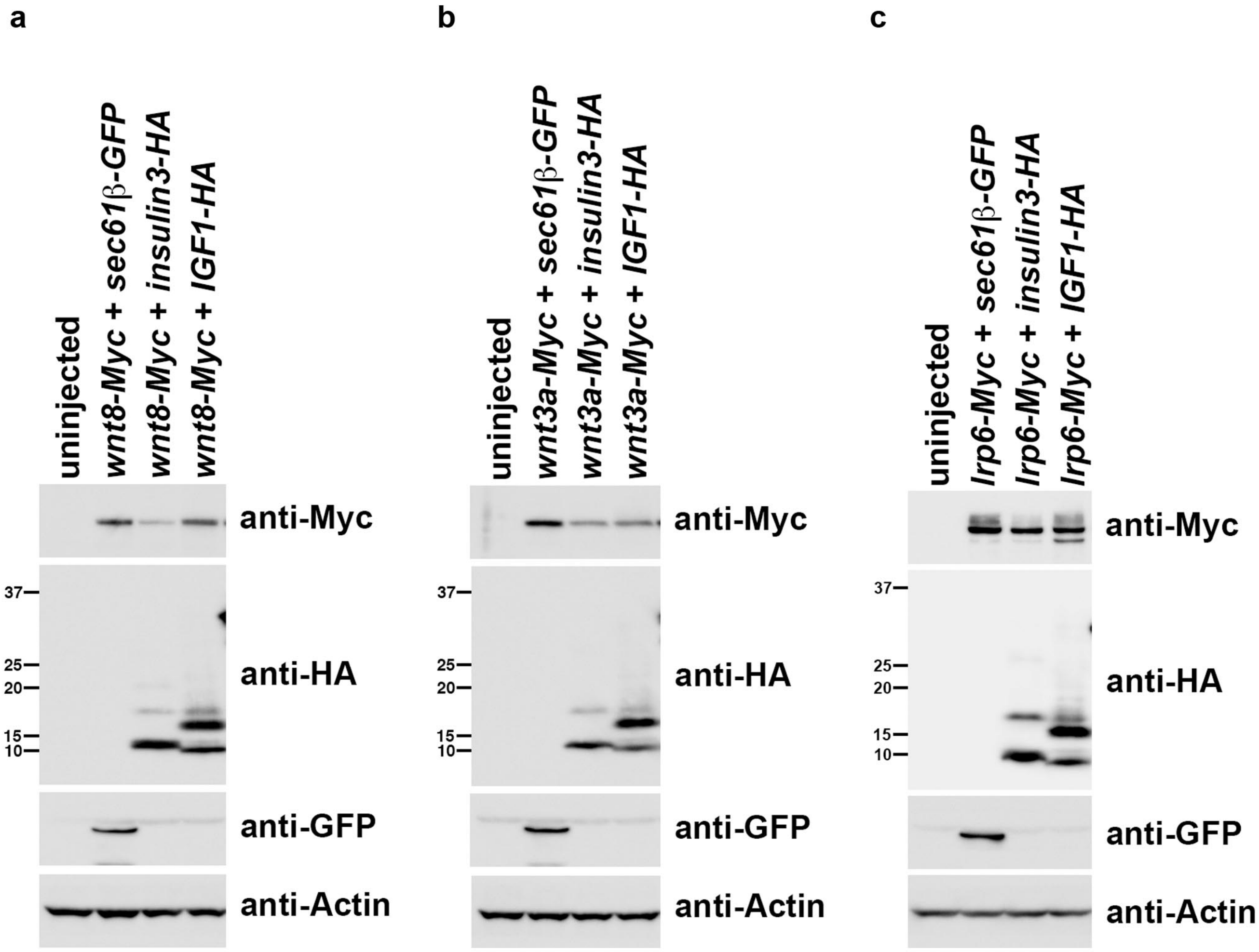

Figure S6. IGF1 has similar activity to Insulin3.
Western blot analysis of embryo lysates. mRNAs was injected into the animal pole of both blastomeres at the two-cell stage in *X. laevis* embryos. Embryos were harvested at stage 11 for western blot analysis. Amounts of mRNA injected per embryos were: *wnt8-Myc* (100 pg), *wnt3a-Myc* (100 pg), *lrp6-Myc* (500 pg), *sec61β-GFP* (1 ng), *insulin3-HA* (1 ng) and *IGF1-HA* (1 ng). (a, b) Insulin3-HA decreased the total amount of Wnt8-Myc (a) and Wnt3a-Myc (b). IGF1-HA also decreased Wnt3a-Myc (b) but not Wnt8-Myc (a). (c) Insulin3-HA decreased levels of the mature form of Lrp6-Myc that localized to the plasma membrane. IGF1-HA had a slight effect on mature form of Lrp6-Myc and increased the non-glycosylated form of Lrp6. Numbers indicate the molecular weight of proteins (kDa).

Fig. 1a

Fig. 1d

Fig. 2a

Fig. 2b

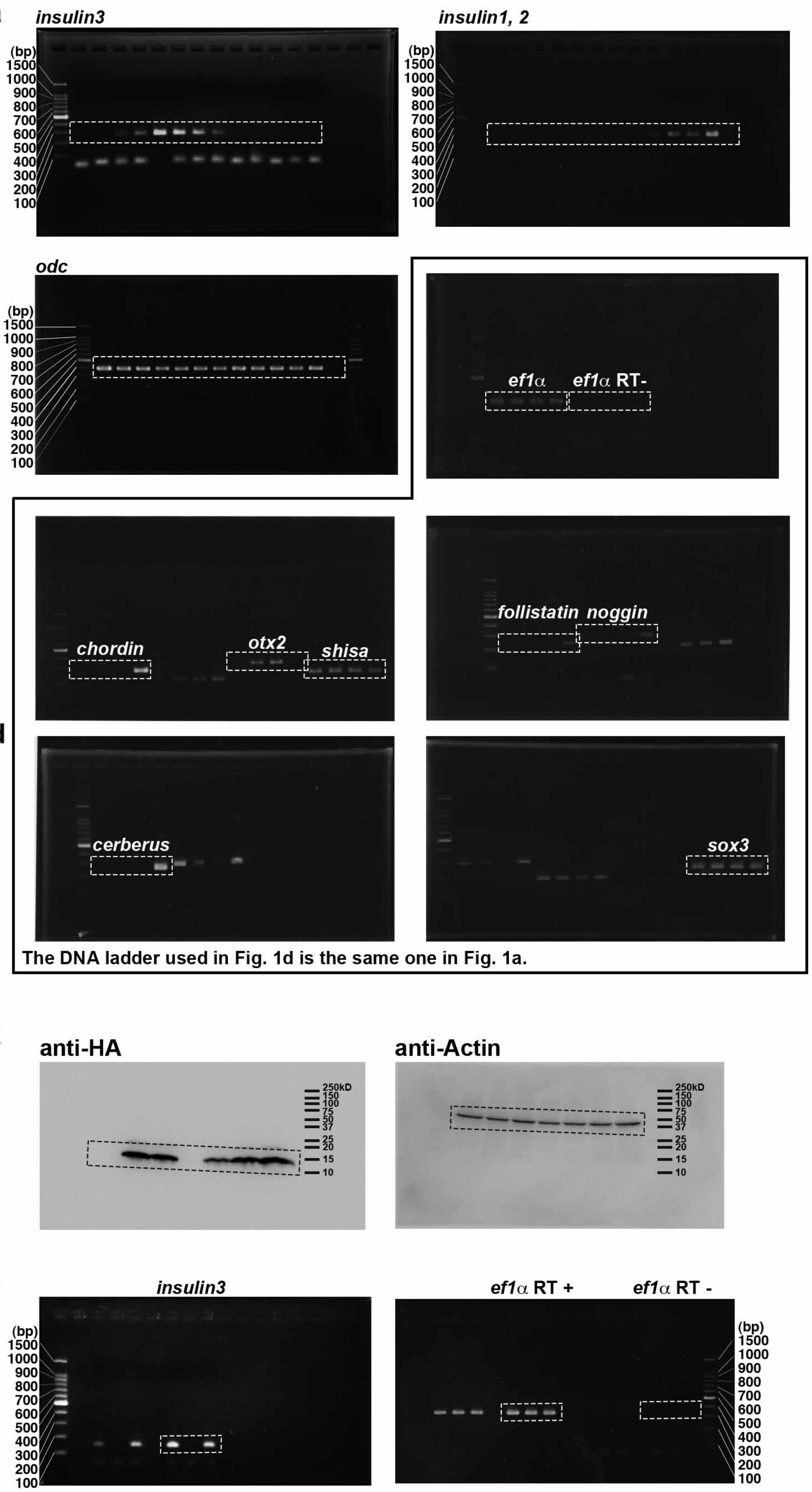

Figure S7. Full-length blots and gels in Figure 1 and 2.
Dashed boxes indicate the portion of the blot and gel included in the figures.

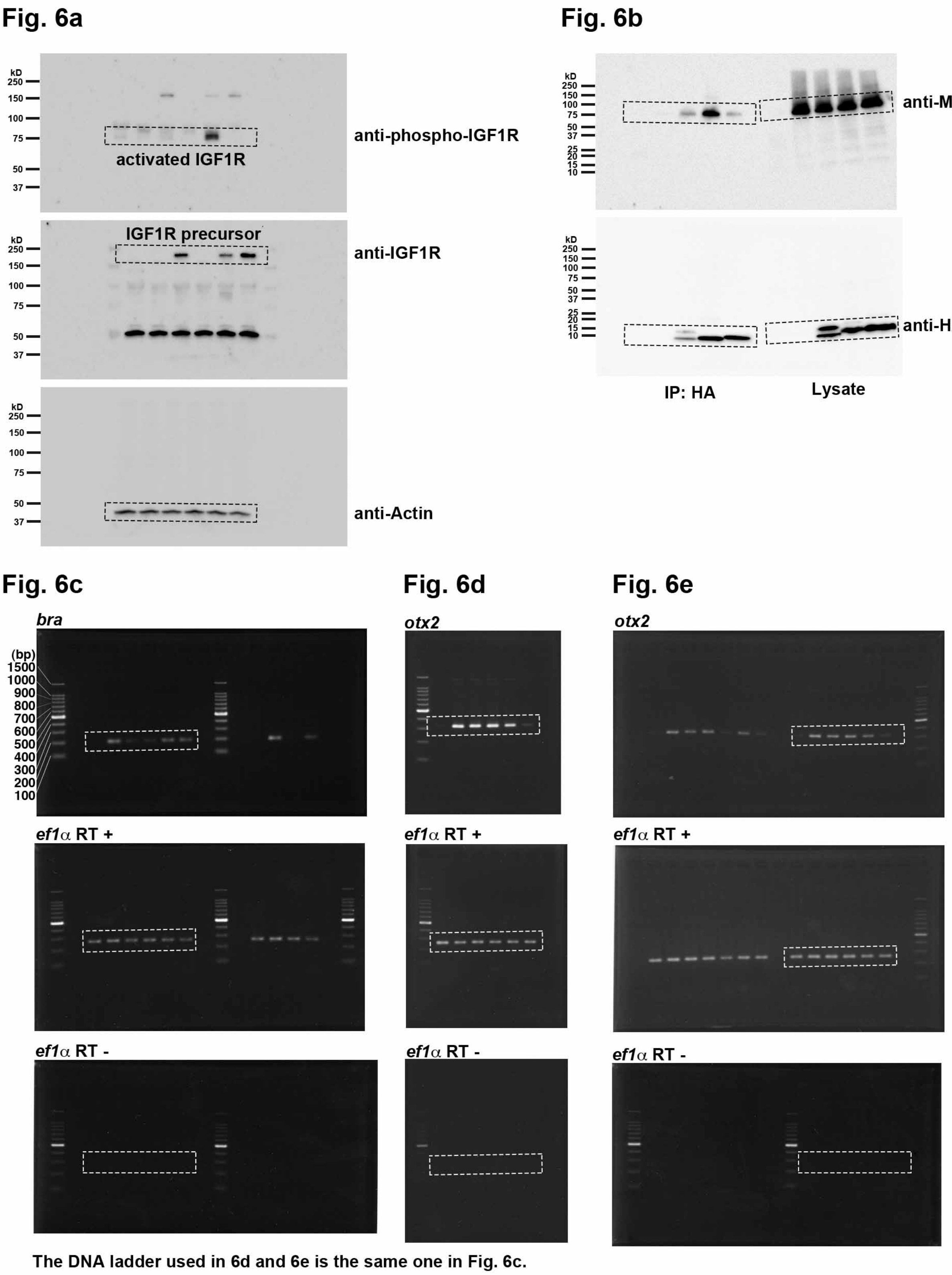

Figure S8. Full-length blots and gels in Figure 6.
Dashed boxes indicate the portion of the blot and gel included in the figures.

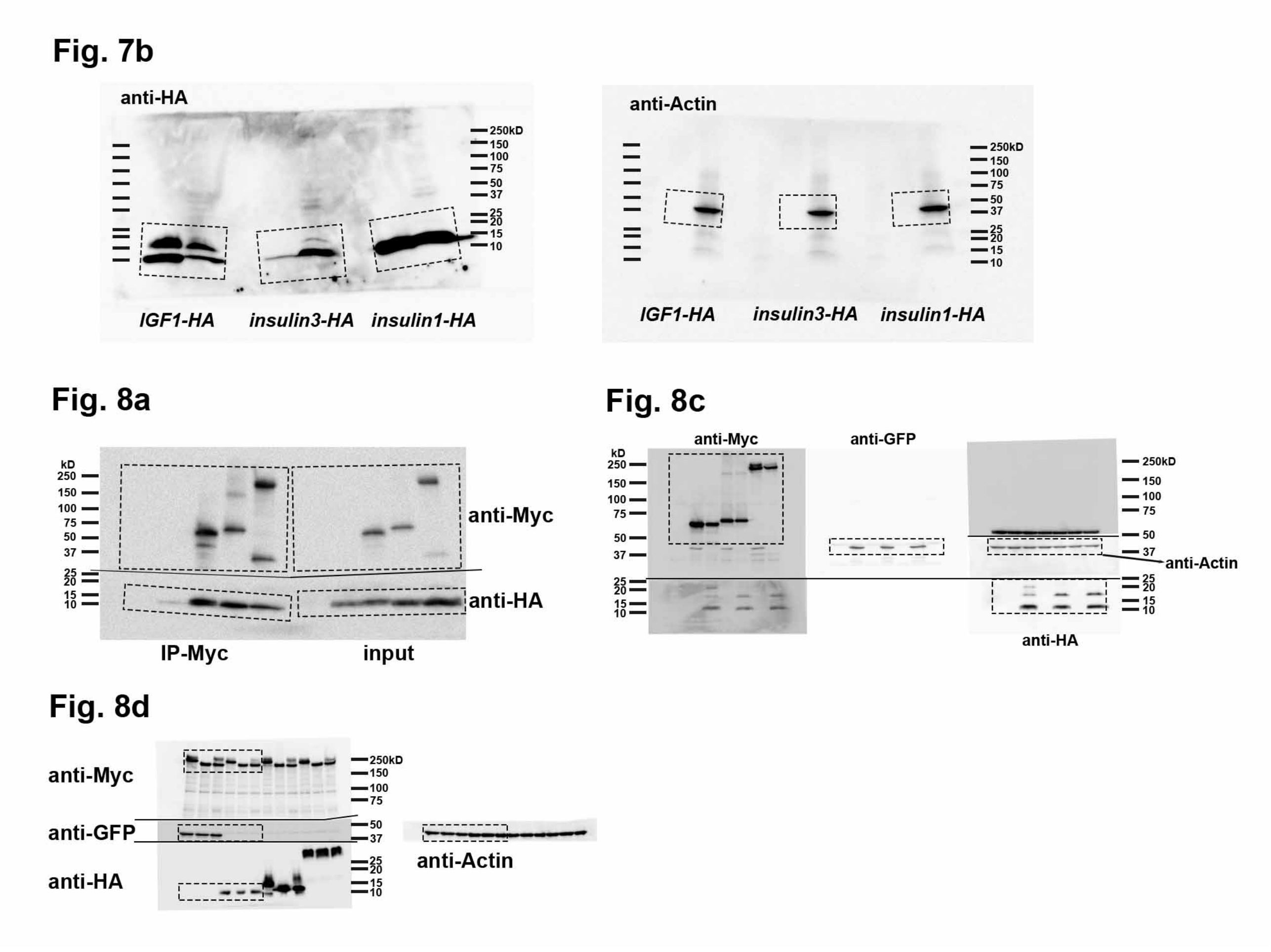

Figure S9. Full-length blots in Figure 7 and 8.

Dashed boxes indicate the portion of the blot included in the figures. The membrane used to generate Figure 8a, 8c, and 8d were cut into two or three sections and developed with different antibodies. The black line indicates the position of the cut.
